# Evaluating a WeChat-Based Health Behavioral Digital Intervention for Patients With Hypertension: Protocol for a Randomized Controlled Trial

**DOI:** 10.2196/46883

**Published:** 2023-09-12

**Authors:** Ting Sun, Huanhuan Zhao, Zenghui Ding, Hui Xie, Linlin Ma, Yu Zhang, Yingying Wang, Yinju Yang, Chunyi Xu, Yining Sun, Xuejie Xu, Zuchang Ma

**Affiliations:** 1 Science Island Branch University of Science and Technology of China Hefei China; 2 School of Nursing Bengbu Medical College Bengbu China; 3 Institute of Intelligent Machines Hefei Institutes of Physical Sciences Chinese Academy of Sciences Hefei China; 4 School of Computer and Information Engineering Chuzhou University Chuzhou China; 5 Sanxiaokou Community Health Service Center Hefei China

**Keywords:** adherence, community health, hypertension, health behavior, mobile phone

## Abstract

**Background:**

Hypertension is the most prevalent chronic condition and a significant risk factor for cardiovascular and kidney diseases. The efficacy of health behavioral interventions in blood pressure (BP) control has been demonstrated by a large and expanding body of literature, with “adherence” playing a crucial role. WeChat is the most common social communication mobile app in China, and it has been shown to be an acceptable delivery platform for delivering health interventions. The WeChat-based health behavioral digital intervention program (WHBDIP) showed high feasibility and efficacy. However, the results regarding BP improvement between the WHBDIP and control groups were inconsistent.

**Objective:**

The objective of this study is to develop a WHBDIP and assess its efficacy in controlling BP and improving adherence among patients with hypertension.

**Methods:**

A 2-arm, parallel-group, and randomized trial design was used. Patients older than 60 years and with hypertension were randomly assigned to either the control group or the experimental group, which received a 12-week intervention. The program, primarily developed based on the Behavior Change Wheel (BCW) theory, offers health education on exercise, diet, BP monitoring, and medicine adherence (MA). It also includes other behavior interventions guided by an intervention manual, incorporating behavior change techniques (BCTs). The primary outcomes encompass BP and adherence indicators, while the secondary outcomes encompass cardiovascular function indicators, body composition indicators, learning performance, satisfaction, and acceptability. The exercise and blood pressure monitoring adherence (BPMA) indicators for the WHBDIP group were assessed weekly via WeChat during the initial 3 months, while other outcome data for both groups will be collected at the baseline assessment phase, 3 months after the intervention, and 1 year after the program.

**Results:**

The trial will assess the efficacy of WHBDIP for patients with hypertension (N=68). The WHBDIP seeks to enhance participants' knowledge of healthy behaviors and assist patients in developing positive health behaviors to improve their health outcomes. Patient recruitment for individuals with hypertension commenced on September 5, 2022, and concluded on September 19, 2022. The 3-month intervention and phased data collection were finalized in January 2023. Data analysis will commence in August 2023, and the final 1-year health outcome results will be collected in September 2023.

**Conclusions:**

A successful WHBDIP will establish the management mode as a feasible approach for hypertension management in the community. Additionally, it will pave the way for the development of related mobile health programs.

**Trial Registration:**

Chinese Clinical Trial Registry ChiCTR2200062643; https://tinyurl.com/mwyv67wk

**International Registered Report Identifier (IRRID):**

PRR1-10.2196/46883

## Introduction

Hypertension, the most prevalent chronic condition and a significant risk factor for stroke, ischemic heart disease, and kidney disease, has become a primary contributor to the global disease burden [1,2]. It is directly responsible for more than 8.5 million deaths worldwide annually [3]. Furthermore, it imposes a substantial economic burden. In China, hypertension accounts for 6.6% of the total national health expenditure [4]. Over the past 30 years, the number of adults with hypertension has risen from 650 million to 1.28 billion [1]. In China, the prevalence of hypertension in adults was 25.2% [5] with awareness, treatment, and control rates at 51.5%, 45.8%, and 16.8%, respectively [6].

Less than half of young patients with hypertension and two-thirds of eligible older patients with hypertension achieve blood pressure (BP) control goals [6]. The low control rate may be attributed to lifestyle-related risk factors, such as poor eating habits and low levels of physical activity [2]. Researchers have found that encouraging patients to follow the recommended health behaviors significantly reduces BP. For example, patients under the supervision of medication taking experienced a 5-mm Hg decrease in daily systolic blood pressure (SBP) compared to unsupervised individuals [7]. Adhering to the Dietary Approaches to Stop Hypertension (DASH) resulted in a 26% reduction in developing high BP [8], and in those already diagnosed with hypertension, SBP and diastolic blood pressure (DBP) decreased by 1.5 and 1.1 mm Hg, respectively [9]. Following various types of recommended exercise led to an SBP reduction of 7.23-8.69 mm Hg [10]. Improving BP monitoring adherence behavior resulted in SBP and DBP reductions of 2.53-4.2 and 1.45-2.4 mm Hg, respectively [11]. High medication adherence, combined with comprehensive interventions such as diet and exercise management, can achieve better BP control effects [12,13]. The results showed a reduction of 12.1 and 26.1 mm Hg in SBP and DBP, respectively, before and after comprehensive health behavior intervention [14], and a decline of 7.5 and 3.9 mm Hg for SBP and DBP, when compared with nonintegrated intervention group [15]. Considering all of this evidence, it appears that comprehensive health behavior intervention can achieve better BP control effects, with adherence being the key.

Digital health (such as mobile health [mHealth] or telehealth) intervention strategies have been used to improve hypertension management outside the hospital. These strategies enhance patient adherence behaviors and increase the feasibility of delivering convenient, continued health interventions in primary health care settings with unequal resource distribution and understaffing. Several recent meta-analyses have demonstrated that using an internet-based interactive intervention mode combining health information with decision support via a computer or mobile phone could lead to a reduction of mean SBP by 2.23-3.74 mm Hg and mean DBP by 1.14-2.99 mm Hg [16-18]. Most of the interventions conducted by the investigators aimed to influence “self-management” or “adherence behaviors” in patients with hypertension [16-18]. Another systematic review of the literature showed the improvement of patient adherence after mHealth intervention [12,17,19-21].

WeChat, a mobile app, is the most prevalent social communication tool in China, with over 1 billion monthly active users of all ages. Approximately 902 million users log into WeChat every day [22]. A substantial amount of prior research in health promotion has focused on WeChat-based programs, which have demonstrated high acceptability, feasibility, and efficacy [23]. WeChat-based health interventions have been found to increase adherence and willingness to seek treatment, improve follow-up rates, enhance health outcomes, quality of life, and self-care ability, and also promote patient satisfaction [23-28]. For hypertension management, WeChat-based programs showed improvements in BP monitoring, diet, self-efficacy, and self-management scores [29,30]. However, the effect of lowering BP is inconsistent. One study reported a significant improvement in BP (SBP: –6.9 and DBP: –3.1 mm Hg) between the intervention and control groups [30], while another study showed no statistical differences between the 2 groups [31]. Therefore, we aim to assess the effectiveness of the WeChat-based health behavioral digital intervention program (WHBDIP) in 2 groups after 3 months of intervention and at the end of an extended additional 7 months. This program involves exercise, diet, BP monitoring, and medication adherence intervention strategies. Our plan is to investigate whether this approach is accessible and effective in improving outcomes for older individuals with hypertension.

## Methods

### Trial Design and Setting

This is a 2-arm, parallel-group, randomized trial that includes a WeChat-based health behavioral digital intervention lasting for 12 weeks. The trial has been conducted at 2 community health centers, both located in Anhui province—1 in Hefei and the other in Bengbu. The primary data collection and evaluation will take place at 3 time points: the beginning of the project, after 3 months of intervention, and 1 year after the project starts.

### Patients

In this trial, patients with hypertension were recruited through phone or verbal invitations from community health centers. Interested individuals were asked for further information by contact staff members. To promote recruitment, relevant physical health assessments were offered for free to candidates, and unqualified candidates were excluded at this stage. Written informed consents were obtained from eligible patients before randomization. The inclusion criteria for the participants were as follows: (1) previous diagnosis of essential hypertension by medical institutions or initiation of antihypertensive drug treatment, (2) older than 60 years of age, and (3) proficiency in using smartphones and the WeChat app. Patients with the following conditions were excluded: (1) prehypertension or not taking antihypertensive drugs; (2) contraindications to exercise, such as serious cardiovascular and cerebrovascular diseases, muscle and joint pain, or inability to participate in exercise as recommended by a doctor; (3) diabetes or nephrosis; (4) participation in other health intervention programs; and (5) not having a sphygmomanometer.

### Sample Size

The sample size was calculated based on the primary outcome of BP. The effect size, widely used to measure treatment effects of continuous outcomes, is calculated as the difference between groups divided by the pooled SD. A previous meta-analysis, comparing mHealth intervention treatments with other traditional treatments, with the primary outcome being BP, reported an effect size of 0.7 [17]. Assuming α=.05, β=.2, and accounting for up to 20% attrition, a sample size of 68 is needed for this study.

### Intervention

#### WHBDIP Development

WHBDIP is a web-based individual telehealth intervention program for patients with hypertension on WeChat, which includes nondrug intervention health education and behavior change strategies. WHBDIP consists of 4 sections: exercise, diet, BP monitoring, and medication adherence behavior interventions, which together constitute a comprehensive health behavior intervention program.

The exercise intervention was based on an intelligent personalized exercise prescription. This prescription has been verified by experts and has been proved by a 1-year real-world clinical research [32]. Before prescribing, health data are collected by an intelligent health promotion system (including networked equipment and a questionnaire system) installed in the community health service center. The intelligent decision-making module of this system generates safe, reliable, and personalized prescriptions according to the complex health conditions of the residents, especially older patients with 1 or more chronic diseases. In WHBDIP, the health education content matching the exercise prescription was compiled into text and then transformed into animation or video to be included in the electronic knowledge base.

The dietary intervention was designed based on the principles of the DASH. After revising the simplified DASH grading diet index score (SDGDIS), videos related to DASH health education were produced and delivered to subjects through WeChat. Subjects were required to upload photos of their 3 meals per day (including a fist for size comparison) within 7 days. The scores (based on the SDGDIS) and qualitative evaluation text were provided to subjects during this period. After 7 days, the subjects and interveners were required to provide their respective scores.

**Figure 1:**
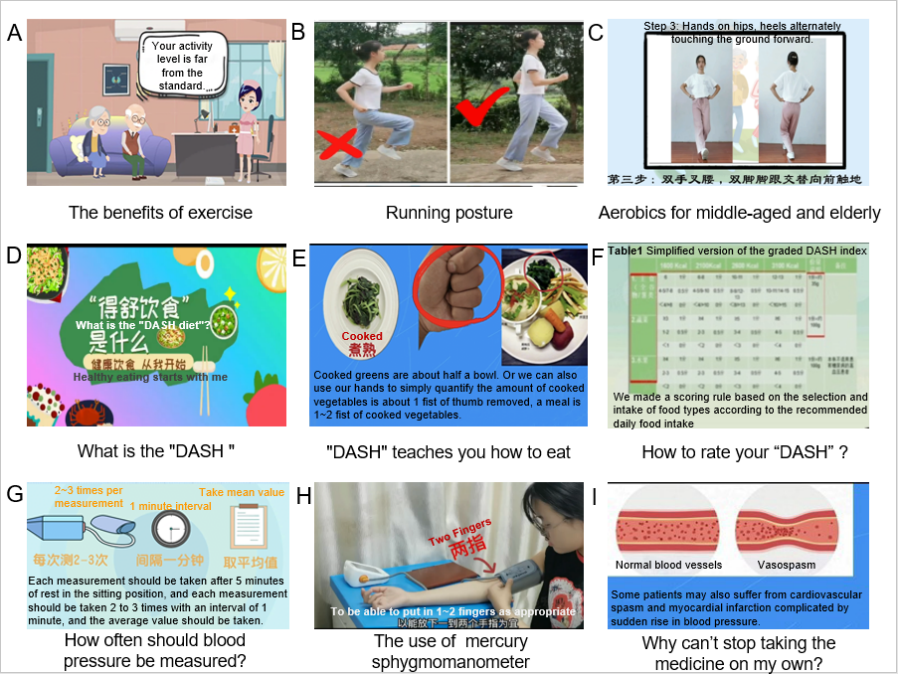
Video screenshots.

For BP monitoring and medicine adherence (MA) intervention, we have created health education videos and used WeChat to send reminders and supervise the health behavior of the subjects. The content of health education related to WHBDIP is displayed in Table 1, and the video screenshots are shown in Figure 1.

**Table 1:** Topics and forms of health education content.

Topic		Form
**Exercise intervention**		
	Intelligent personalized exercise prescription	Pictures and text
	The benefits of exercise	Video
	The difference between aerobic and anaerobic exercise	Video
	The choice of exercise intensity	Pictures and text
	Seated strength training for the older	Video
	Running breath	Video
	Running posture	Video
	When should stop exercising	Video
	Aerobics for middle-aged and older	Video
	Stretching	Video
**Dietary intervention**		
	What is the “DASH”^a^	Video
	“DASH” teaches you how to eat	Video
	How to rate your “DASH”?	Video
**BP^b^ monitoring**		
	How to choose a sphygmomanometer	Video
	How often should BP be measured?	Video
	How much does BP drop to reach the target?	Video
	The use of mercury sphygmomanometer	Video
	The use of upper arm electronic sphygmomanometer	Video
	The use of wrist electronic sphygmomanometer	Video
**MA^c^ intervention**		
	Why can't stop taking the medicine on my own?	Video

^a^DASH: Dietary Approaches to Stop Hypertension.

^b^BP: Blood pressure.

^c^MA: Medicine adherence.

To promote adherence behavior change, we adopted the behavior change wheel (BCW), which is a systematic framework for selecting 25 behavior change techniques (BCTs) from Michie et al's [33] taxonomy that correspond to the 9 intervention functions (shown in Table 2). The use of BCTs was documented in the intervention manual.

**Table 2:** Implementation of BCTs^a^ following Michie et al’s [33] BCW^b^ and taxonomy.

Intervention functions and BCTs		Examples
**Education: increasing** **knowledge or understanding**		
	4.1 Instruction on how to perform the behavior	Provide healthy educational content on how to exercise and eat
	5.1 Information about health consequences	Tell the intervention the benefits of exercising
	4.3 Reattribution	If the subjects attributed their lack of exercise to frequent bad weather, intervenor indicated that the “real” reason might be the person's lack of motivation
**Persuasion: using** **communication to induce positive or negative feelings or stimulate action**		
	15.3 Focus on past success: advise to think about or list previous successes in performing the behavior	Suggest the subjects to think about or list the successful experience of a certain behavior (quit smoking and drinking) before to convince him that he can also develop the habit of scientific exercise or diet
	15.1 Verbal persuasion about capability	Tell the subjects that they can successfully increase their physical activity
	13.5 Identity associated with changed behavior	Ask the subjects to articulate their new identity as “ex-inactive person”
**Incentivization: creating** **an expectation of reward**		
	10.2 Material reward (behavior)	Arrange for the subjects to receive WeChat red packet (money)
	10.6 Nonspecific incentive	Indicates a happy mood for completing the task, such as instructing the subjects to imagine that he has successfully completed today's exercise task, sweating profusely, and feeling happy
Coercion: creating an expectation of punishment or cost		
	—^c^	—
**Training: imparting** **skills**		
	8.1 Behavioral practice or rehearsal	Ask subjects to continue to rate their diet for 7 days
	15.4 Self-talk	Encourage the subjects to use talk to themselves (aloud or silently) before and during exercise to encourage, support and maintain action, like “I Can!”
Restriction: using rules to reduce the opportunity to engage in the target behavior (or to increase the target behavior by reducing the opportunity to engage in competing behaviors)		
	—	—
**Environmental restructuring: changing the physical or social context**		
	12.6 Body changes	Remind the subjects to stretch before and after exercise (except health care type) to prepare their bodies
	12.2 Restructuring the social environment	Encourage the subject to invite family members or friends to exercise together
	3.1 Social support	
Modeling: providing an example for people to aspire to or imitate		
	—	—
**Enablement: increasing** **means or reducing barriers to increase capability (beyond education and training) or opportunity (beyond environmental restructuring)**		
	1.1. Goal setting (behavior)	Determine the weekly exercise time based on exercise prescriptions
	2.7 Feedback on outcomes of behavior	Feedback on diet score and comprehensive evaluation
	2.4 Self-monitoring of behavior	Provides a subjective method to assess exercise intensity and allows subjects to self-monitor
	1.9 Commitment	Sign a contract with subjects that they can do healthy behavior
	1.4 Action planning	Prompt planning of a particular physical activity (running) at a particular time (before work) on certain days of the week
	1.5 Review behavior goals	Examine how well subjects’ performance corresponds to agreed goals such as exercise 30 minutes at a time
	1.6 Discrepancy between current behavior and goal	Compare the difference between the real exercise volume and the target volume
	7.1 Prompts or cues	Send medication reminder to subject
	8.3 Habit formation	Ask the subjects to check in on sports and upload photos of their diets every day
	7.3 Reduce prompts or cues	Reduce medication reminders
	1.2 Barrier identification or problem solving	Analyze the reasons (information are collected by questionnaire) for impeding movement and provide solutions (according to intervention manual)
	4.2 Information about antecedents: advise to keep a record of situations or events occurring prior not to exercise	Record the reasons for not exercising
	11.2 Reduce negative emotions	Instruct the subject to perform relaxation exercises in situations when exercise brings about negative emotions

^a^BCTs: behavior change techniques.

^b^BCW: behavior change wheel.

^c^Not available.

#### Validation of WHBDIP

The WHBDIP (health education content and intervention plan) has been validated through a 2-round Delphi survey by a panel of experts. The panelists consisted of 15 experts, including 4 nutritionists and 1 professor of nutriology, 2 clinical cardiologists, 3 clinical nurses or nursing teachers, and 5 exercise-related experts. All of them held a bachelor’s degree or higher and had at least 5 years of work experience in their respective fields. The panelists were divided into 3 groups to evaluate the content’s appropriateness and relevance for exercise, diet, and BP monitoring or MA management. The content validity index (CVI) was calculated using item-level content validity index (ICVI) and rated on a 4-level scale. In the first round, the ICVI ranged from 0.6 to 0.8. Based on the experts' comments, the content was modified. In the second round, the ICVI reached 1.

### Intervention Procedure and Monitoring

Prior to the experiment, we conducted a physical examination on the subjects at the intelligent health hut and used the system to generate intelligent exercise prescriptions. Health behavioral digital intervention was provided to the experimental group by 5 trained interventionists through WeChat. The interventionists sent health education materials, specific text (such as reminders to take medicine or exercise), or electronic questionnaires according to the intervention manual's procedures. They collected data or pictures about exercise, diet, self-measured BP, and medication every day during the initial 3 months. The control group received routine health interventions, including oral health education about printed exercise prescriptions, dietary guidance, BP monitoring, and the importance of regular medication. After the program, they will have free access to health education electronic materials.

### Randomization, Allocation, and Blinding

This study followed a process of randomization. Before randomization, an individual not involved in subject recruitment and data collection used the SPSS software (version 23; IBM Corp) to generate 68 random numbers and assigned them to either 1 (experimental group) or 2 (control group) using visual binning. These 68 sets of random numbers were printed separately and sealed in individual envelopes. After recruiting a participant, the facilitator opened the envelopes sequentially, and the number found inside each envelope determined the group to which the particular participant belonged. In this study, we blinded data collectors both before and after the trial.

### Ethics Approval

This study received approval from the Ethics Committee of Bengbu Medical College (2022-103) in June 2022. Informed written consent forms were obtained from parents with hypertension prior to the start of the study. All the participants agreed to the anonymous use of their data. Upon completing the intervention and data collection at the community health center, the participants were provided with 50 RMB (based on the current exchange rate of US $1=7.29￥, here it is about US $6.86) worth of daily necessities.

### Outcomes

#### Primary Outcomes

BP will be measured using a cardiovascular function monitor (BX-CFTI-100, Institute of Intelligent Machines) at 3 time points: the first baseline assessment phase (T0), the 12-week end point assessments phase (T1), and the end of the 1-year program (T2). The assessment details have been published elsewhere [32].

Exercise adherence (EA), DA, MA, and BPMA indicators were collected once a week through WeChat for the intervention group until T1. The BPMA will also be collected at T0, T1, and T2 for the control group. The exercise adherence indicator was calculated as the ratio of actual exercise time that meets the prescription's recommended intensity to the total time of the prescription-recommended intensity. PAT will be measured as an indicator by questioning subjects in both groups at T0, T1, and T2 about the type, duration (in minutes), and frequency (per week). It will be measured using the minutes or metabolic equivalent per week. The MA indicator will be measured at T0, T1, and T2 for both groups using the Modified Morisky Scale (Chinese version-MMS-8, Certificate Number: 8538-1877-1559-6025-5310) [34-36], which includes 8 simple questions. The rating is “poor medication adherence” for a final total score of <6 points, “moderate medication adherence” for a score of 6-7, and “good medication adherence” for a score of 8. Dietary adherence (DA) will be assessed at T1 and T2 for both groups based on their compliance with the “types of diet” and the “recommended amount of diet” (3 response levels for each question).

#### Secondary Outcomes

Cardiovascular function indicators and body composition indicators will be assessed at T0, T1, and T2 in the community health center. Specific details have been published elsewhere [32]. Learning performance of health education knowledge will be evaluated using a comprehensive self-made questionnaire about the material at T0 and T1. Satisfaction and acceptability of this program were assessed at T1 for the intervention group through face-to-face interviews using 3-5 semistructured questions. The questions included: (1) “Do you find the program useful?” (2) “Is it worth your time?” and (3) “Would you feel confident recommending this program?” These questions were used in previous studies [37].

### Data Collection and Management

**Figure 2:**
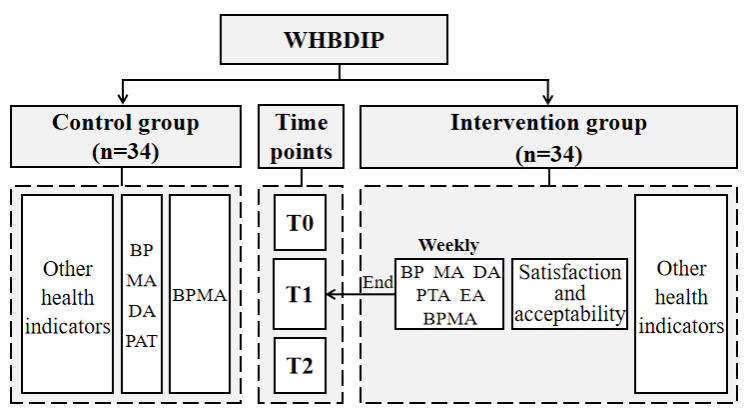
Figure 2. Study flowchart. BP: blood pressure; BPMA: blood pressure monitoring adherence; DA: dietary adherence; EA: exercise adherence; MA: medicine adherence; PAT: physical activity time; WHBDIP: WeChat-based health behavioral digital intervention program.

Health-related data will be collected and entered into the intelligent health promotion system at T0, T1, and T2. During the intervention, data for the intervention group were collected using WeChat (as shown in Figure 2). An independent data manager checked the database every week to ensure its integrity. Data lockup is being implemented to prevent any postmodification.

### Statistical Analysis

All analyses will be performed using SPSS. Continuous variables will be presented as mean and SD values. Categorical variables will be expressed as numbers and percentages. Independent samples *t* tests and chi-square tests will be conducted to assess the significance of differences in baseline characteristics or outcomes between the control group and intervention group at 3 time points: before the start of the intervention, 12 weeks after the intervention, and 1 year after the program. Paired *t* tests and McNemar's test will be used to compare the differences in some outcomes before and after the 3-month intervention, as well as before the trial and after the end of the program. A *P* value of less than .05 will be considered statistically significant.

## Results

The study was funded in December 2021. The WHBDIP trial was approved by the Ethics Committee of Bengbu Medical College in June 2022 (2022-103) and registered in August 2022 (ChiCTR2200062643). Patient recruitment for those with hypertension started on September 5, 2022, and was completed on September 19, 2022. The 3-month intervention and phased data collection were completed in January 2023. Data analysis will commence in August 2023, and the final 1-year health outcome results will be collected in September 2023. We expect the primary outcomes to show changes in adherence indicators during the 3-month intervention, and health outcomes, including BP and other cardiovascular indicators, to change after the intervention and 1 year later. Furthermore, we will report on dropout rates and reasons for dropout during the trial.

## Discussion

The WHBDIP is a WeChat-based digital health program that aims to improve the self-management of BP and promote adherence behavior change among patients with hypertension. If the effectiveness of WHBDIP can be verified, it may be attributed to several key characteristics. First, all interventions were developed based on robust evidence, and we further optimized them. Similar to DASH, a proven effective diet for patients with hypertension, we have created a map of food portions tailored to the dietary characteristics of Chinese people and included beverage options in the scoring table. Second, WHBDIP is a theory-based intervention tailored to patients with hypertension. We specified the target behavior (adherence) and conducted a scientific and systematic procedure to identify the appropriate functions from the 9 behavior functions. We then selected the corresponding BCTs from Michie et al’s [33] taxonomy based on the BCW. These BCTs were compiled into the intervention manual, which will serve as the foundation for the development of the mHealth intervention program. Third, we provided tailored interventions, including intelligent personalized exercise prescriptions and different intervention contents for adherence. For example, people with different movement barriers require different persuasion techniques. Fourth, we provided one-to-one web-based health services, which could increase participant satisfaction. Fifth, this trial involved health-related professionals and followed a community-based telehealth intervention approach. Finally, we designed this trial to meet methodological demands, including adequate power, allocation concealment, and necessary blinding.

If the WHBDIP is proven to be effective, it could be integrated into the upcoming eHealth promotion program. We will develop community-based apps to promote the management of patients with hypertension, including patient terminals and health service provider terminals. The app will deliver personalized e-interventions, such as reminders, videos, and other content, to patients through specific logic that incorporates behavioral change techniques. This will enable the manual intervention to be automated. It may serve as an alternative, convenient, and cost-effective approach, deserving further experimental verification, for doctors and nurses in primary health care institutions to provide tailored and consistent health services for patients with hypertension.

The study has some limitations. First, due to the nature of the intervention, interventionists in this trial cannot be blinded. To reduce the impact, we provide detailed training and an intervention manual to minimize the subjective influence of interventionists. This way, interventions can be standardized as much as possible. Second, some outcome measures rely on patient self-report scales, which may somewhat affect the reliability of the results.

## Abbreviations

BCT: behavior change technique
BCW: behavior change wheel
BP: blood pressure
BPMA: blood pressure monitoring adherence
CVI: content validity index
DA: dietary adherence
DASH: Dietary Approaches to Stop Hypertension
DBP: diastolic blood pressure
EA: exercise adherence
ICVI: item-level content validity index
MA: medicine adherence
mHealth: mobile health
PAT: physical activity time
SBP: systolic blood pressure
SDGDIS: simplified DASH grading diet index score
WHBDIP: WeChat-based health behavioral digital intervention program

## Data Availability

Data sharing is not applicable to this paper, as the trial is not finished. The data that will be collected at the end of the trial will be described in the next paper, which will be published in a future paper when the data become available.
